# The AMEL study, a cross sectional population-based survey on aging and malnutrition in 1200 elderly Lebanese living in rural settings: protocol and sample characteristics

**DOI:** 10.1186/1471-2458-13-573

**Published:** 2013-06-12

**Authors:** Christa Boulos, Pascale Salameh, Pascale Barberger-Gateau

**Affiliations:** 1 Department of Nutrition, Faculty of Pharmacy, Saint Joseph University, Beirut, Lebanon; 2 Faculty of Public Health, Lebanese University, Beirut, Lebanon; 3 ISPED, Centre INSERM U897-Epidemiologie-Biostatistique, Univ. Bordeaux, Bordeaux F-33000, France; 4 INSERM, ISPED, Centre INSERM U897-Epidemiologie-Biostatistique, Bordeaux F-33000, France

**Keywords:** Malnutrition, Nutritional status, Elderly, Aging, Rural health

## Abstract

**Background:**

Lebanon is faced with a particular challenge because of large socioeconomic inequality and accelerated demographic transition. Rural residents seem more vulnerable because of limited access to transport, health and social services. No information is available regarding health, nutrition and living conditions of this specific population. The purpose of the AMEL (Aging and Malnutrition in Elderly Lebanese) study is to assess the nutritional status of community dwelling elderly people, aged 65 years and above, living in a rural settings in Lebanon, in line of socioeconomic factors, health and living conditions. The present paper will describe the gender specific characteristics of the study population.

**Methods:**

AMEL is a cross-sectional population based study conducted between April 2011 and April 2012 including 1200 elderly individuals living in the 24 rural Caza (districts) of Lebanon. People aged greater than or equal to 65 y were randomly selected through multistage cluster sampling. Subjects were interviewed at their homes by trained interviewers. The questionnaire included the following measures: socio-demographic factors, nutritional status (Mini Nutritional Assessment, MNA), health related characteristics, functional ability, cognitive status, mood and social network.

**Results:**

The sample included 591 men (49.3%) and 609 women (50.8%). Mean age was 75.32 years and similar between genders. Malnutrition (MNA < 17) and risk of malnutrition (MNA between 17 and 23.5) were present in 8.0% (95%CI 4.9%-11.1%) and 29.1% (95%CI 24.0%-34.2%) respectively of the participants, and more frequent in women (9.1% and 35.3% respectively). Regarding socio-demographic status, among women the level of illiteracy and poor income was significantly higher than in men. Moreover, chronic diseases, poor self perceived health, frailty, functional disability, depressive symptoms and cognitive impairment were particularly high and significantly more frequent in women than in men.

**Conclusion:**

The present study provides unique information about nutritional status, health and living conditions of community dwelling rural residents of Lebanon. These findings may alert policy makers to plan appropriate intervention in order to improve the quality of life and increase successful aging.

## Background

With the growing number of elderly people, chronic diseases and disability become a public health challenge especially in developing countries, where the health care sector is less developed and suffering from limited resources [1]. Moreover, the elderly population in developing countries is growing more rapidly than in more developed nations and it is projected that in 2020, 70% of those aged above 55 y will live in developing countries [2]. This accelerated demographic transition does not give enough time to allow these countries to develop their health, economic and social infrastructures in order to deal with the emerging older population. Another concern is that population aging in the developing world is accompanied by persistent poverty [3]*.* Gro Harlem Brundtland, former Director-General of the World Health Organization stated: “*While the developed countries became rich before they became old, the developing countries will become old before they become rich*” [4].

Living in rural areas in developing countries may carry an additional disadvantage. Indeed, several studies reported that elderly people living in rural areas suffer from worse health conditions, are less educated and have lower incomes compared with those living in cities [5-7]. They face specific problems including limited access to transport, facilities and health or social services. Nevertheless, rural residents may receive more community support [8], have a healthier lifestyle, more physical activity as well as healthier food habits which may counterbalance some of the disadvantages otherwise mentioned [9].

Nutritional status of older people results from a complex interplay between dietary, socio-economic, physical and psychological factors [10]. In addition, malnutrition (in form of under nutrition) or overweight will limit the ability to move, perform daily activities, and worsen comorbidities. Thus, nutritional status is a key factor in maintaining health and autonomy, especially when resources and health care are sparse.

Lebanon, a small middle-eastern country and one of the first among the Arab countries to be affected by the demographic transition, has currently the highest percentage of elderly aged 65 years and above (7.4%) among Arab countries. This proportion may reach 10.2% in 2025 and 19.3% of the population in 2050 [11,12]. One of the major problems this growing population is faced with is the lack of policies and retirement pensions [11], the high cost of health insurances as well as health disparities [5,6]. These facts as well as high illiteracy [13] and poverty [14] mainly present in rural areas, may affect health status and contribute to the growing vulnerability of older individuals. Unfortunately, little is known about the characteristics and the needs of these elderly people. Most studies carried out among Lebanese older adults focused on nursing home populations [15,16] or on elderly people living in refugee camps and in underprivileged communities [17,18].

The AMEL (**A**ging and **M**alnutrition in **E**lderly **L**ebanese) study was designed to assess the nutritional status of community dwelling elderly people aged 65 years and above, living in rural settings in Lebanon, in line of their health and living conditions, socio-economic factors and dietary habits. This article describes the gender specific characteristics of the study population including demographic, socio-economic conditions, health and functional status as well as nutritional assessment. Dietary habits were not presented in this paper, but will be the subject of a second paper in preparation, along with factors associated with nutritional status.

We underline the fact that the term of “malnutrition” used in this manuscript refers to “under nutrition”.

To our knowledge, this is the first study investigating the living and health conditions of community dwelling elderly Lebanese living in rural areas.

## Methods

### Study design

We carried out a cross sectional population based study which enrolled 1200 community dwelling elderly individuals aged 65 years and above, living in rural areas of seven out of the eight Mohafazat in Lebanon.

### Sample size calculation

The sample was selected through multistage cluster sampling. The sample size has been established according to the prevalence of home-living elderly people, either suffering from malnutrition or considered at risk of malnutrition (average 40%). These results are based on a review of 48 studies including either healthy or frail elderly individuals [19] and a retrospective study of pooled data [20] based on the Mini-Nutritional-Assessment (MNA) [21]. Although some of the study samples were of convenience nature and mainly focused on European population, we considered these results as the most appropriate for our sample calculation, as representative data from developing countries are lacking. A minimum of 1024 subjects was necessary to establish a 95% confidence interval, with an accuracy of +/− 3%, taking clustering into account. Due to possible missing values in several items, a final sample size of 1200 elderly was chosen.

### Subjects and setting

Lebanon is divided into eight Mohafazat (governorates); each of them consists of several districts (Caza) forming a total of 25 Caza. As our study included only rural elderly subjects, the Caza of Beirut (urban area) was excluded. In each of the remaining 24 Caza (stratum), two villages were randomly selected from the list of villages provided by the Central Agency of Statistics in Lebanon, except for two Caza where only one large village was selected giving a total of 46 villages [22]. Within each village, a random sample of 25 elderly individuals was drawn from the small villages and 50 from the larger villages, based on the list of households provided by the municipality or other local authority. A replacement list was prepared in case of absence or refusal of participation. The inclusion criteria were: to be at least 65 years old, to live at home in rural areas, to be free from terminal illness and not tube fed. The study received the approval of the ethics committee of St Josephs University of Beirut.

### Questionnaire

The study was based on a comprehensive multi-component questionnaire, administrated by trained interviewers, including the assessment tools as described below. The questionnaire was translated back and forth from French to Arabic by two persons fluent in both languages. People were questioned after oral consent at their place of residence. Written consent was not considered necessary because it was an observational study. Also, participants remained anonymous and individual results were kept confidential. A pilot study including 100 individuals was performed previously in order to pretest the feasibility of the questionnaire. According to the results, some minor changes were made. The interview duration of 40–50 minutes was considered acceptable. If the participant was unable to answer, the help of a family member was required. The main survey was conducted over a period of 12 months between April 2011 and April 2012.

#### Assessment tools

### Socio-demographic factors

The variables recorded included demographic characteristics (age, gender, marital status, village of residence) and living conditions (living alone or with others). Information about the financial situation was recorded by two questions: one question regarding the personal monthly income which, according to the national minimum wages, was categorized into < 300.000 LL (~200 USD), 300.000 – 600.000 LL and > 600.000 LL (~400 USD). According to a survey conducted by the Central Administration of Statistics of Lebanon in 2004, about 8,7% of Lebanese adults live below the extreme poverty line corresponding to an annual income of 2400 USD (200 USD monthly) per household [23]. The second question asked about financial dependency from children, with answers ranging between “totally dependent, partially dependent or independent”. Information about educational level was obtained using the following categories: illiteracy, less or at least 8 years schooling (primary school), less or at least brevet (middle school), less or at least high school graduation and university level. Regarding the main occupation, individuals were questioned about the longest occupation held, which was categorized into: farmer, employee or manager, self-employed, without work (including housework); they were also asked if they were still working. The question about health insurance was categorized into three groups: without insurance, private insurance, national social security fund (NSSF) and others.

### Anthropometric measures and nutritional status

Weight was taken in light indoor clothes without shoes by electronic digital scale to the nearest 0.1 kg, whereas height was measured in standing position to the nearest 0.1 cm. Body Mass Index (BMI) was computed as [weight (kg)/height (m^2^)]. Nutritional status was assessed by the MNA in its Arabic version [24]. The MNA, developed by Guigoz et al. [25], is the most established, best validated and widespread nutritional assessment tool used among geriatric population [26]. It has been translated into more than 20 languages and is cited in nearly 200 publications. The MNA includes 18 questions regarding anthropometric, general, dietetic, and subjective assessment. Based on the total score, subjects were classified into three categories: malnourished (<17), at nutritional risk (17 ≤ score < 24), adequate nutritional status (≥ 24) [25].

### Health characteristics

Health related characteristics were assessed by self related health status (SRH) based on a 5 item scale. This measure has shown to be a reliable indicator for overall health status [27] in developed countries but also in most Arabic countries [28]. Comorbidities were recorded by asking participants if they suffered from chronic physician-diagnosed conditions such as hypertension, diabetes, etc. Drug intake was estimated by the number of drugs taken daily on a regular basis as prescribed by a physician and checked with packages shown to the interviewer. Furthermore, the participants were questioned about chronic pain (yes/no), defined as feeling pain for at least 3 months, insomnia (no or occasionally/often or always) as well as recent hospitalization (<1 year). Frailty was assessed by the SOF (**S**tudy of **O**steoporotic **F**ractures) frailty Index [29], which included 3 items: involuntary weight loss for one year, inability to rise from a chair without using arms and reduced energy level for at least 3 days during the past week. Based on the original SOF frailty index, frailty status was defined as robust (0 component), pre-frail (1 component), and frail (2 or more components). Oral health assessment included three dichotomous questions about chewing problems, total or partial loss of dentition and wearing dental prosthesis. Finally, tobacco use was estimated by asking about current smoking, whereas the level of physical exercise was assessed by estimating the frequency and duration of exercise (excluding housework) by “a minimum of 30 min daily”, “occasionally” or “never”.

### Functional status

Functional status was investigated through the Activities of Daily Living (ADL), a 5-item scale commonly used in comprehensive geriatric assessment evaluating the basic activities like bathing, toileting, clothing, walking and eating by his/her own [30]. This scale was validated in its Arabic version by Nasser & Doumit [31] in a sample of Lebanese elderly living in nursing homes. Continence was not considered in this scale because difficulties in bladder or bowel control reflect an abnormality in a particular physical system and should therefore be considered as impairment rather than a disability [32]. According to several authors [33,34], we defined three main groups: “not disabled” was defined as independent in all 5 ADL, “moderately disabled” as dependent in one or two items, “severely disabled” as dependent in three or more items. The Instrumental ADL (IADL) included four activities: telephone use, use of transportation, responsibility for drugs intake and budget management. This 4 item IADL scale has been shown to be associated with cognitive impairment in community dwelling elderly subjects [35]. Individuals were considered as fully independent (coded 0) if they could perform the IADL item without any help, otherwise they were considered partially dependent (coded 1). The final score, ranging from 0 to 4, was computed by summing the number of IADL dependencies for these four items. Subjects without telephone (n = 288) as well as subjects free of drug intake (n = 152) were not considered in the analysis. Balance disorder was assessed by the “one-leg balance” test, a useful screening tool of fall risk among elderly individuals [36]. Subjects were asked to stay on one leg without using their arms. Moreover, participants were asked if they experienced one or more falls during the past year.

### Psychosocial and cognitive status

Mental status was assessed by the 5 item Geriatric Depression Scale (GDS-5), a dichotomized 5- item scale (score ranges from 0–5) allowing to detect depressive disorder in elderly people [37]. Presence of depressive symptoms was defined as a score of two or above [37]. The 5-item WHO Well Being Index [38] was used to assess the mood of our study sample, as this instrument had previously been validated in the Arabic version by Sibai et al. [38] among Lebanese elderly. The WHO-5 Arabic version allows the detection of depression among Lebanese elderly at a cut-off point less than 13 [38]. Cognitive status was assessed by the Mini-mental-state (MMS) examination [39], the most commonly used screening tool for cognitive impairment worldwide. In order to take into account the high level of illiteracy, we constructed a modified version adapted to illiterate subjects (MMS 2), whereas the original MMS (MMS 1) translated in Arabic language was used for literate elderly. In the MMS 2 form, question 28 including a written order (“Close your eyes”) was replaced by the same order but given verbally (the interviewer asks the subject to close the eyes). Furthermore, regarding question 29, where the patient is required to write a sentence, the participant was asked to construct a sentence orally including a subject, a verb and an object. As no cut-off points were defined in Lebanese elderly, the results were divided into quartiles. To investigate the social network we used the Lubben Social Network Scale 6 (LSNS 6) as described by Lubben et al. [40]. This tool is an abbreviated version of the original LSNS scale [41], which was especially developed for elderly populations and has been shown to be associated with a wide range of health indicators. The LSNS 6 is based on 3 questions assessing the family network, as follows*: “How many relatives do you see or hear from at least once a month? How many relatives do you feel close to such that you could call on them for help? How many relatives do you feel at ease with that you can talk to about private matters?”* These same questions are repeated by replacing the word “relatives” with the word “friends”. The answers were as follows: none (coded 0), one (coded 1), two (coded 2) three or four (coded 3), five to eight (coded 4), nine or more (coded 5). The total score is the sum of the 6 items, ranging from 0 to 30. According to the author [40], at a score below 12, the person is considered as at risk for social isolation. Subjective loneliness was assessed by the modified version of the Jong- Gierveld Loneliness Scale as described by Wilson et al. [42]. This 5 item scale included the following: *“I experience a general sense of emptiness,” “I miss having people around,” “I feel like I don’t have enough friends,” “I often feel abandoned,” and “I miss having a really good friend”.* As Wilson [42], we used a dichotomous scale where “*yes*” was scored for 1 and “*no*” for 0. Following the authors’ instruction, higher values indicated more loneliness.

### Statistical analysis

The Statistical Package for Social Sciences (SPSS) version 19.0 was used to enter and analyze data. Cluster effect was taken into account when computing confidence intervals, according to Rumeau-Rouquette et al. [43]. Percentages were used to present nominal variables, while means and standard deviations were applied for presenting continuous variables. The Chi Square test was used for cross tabulation of qualitative variables in bivariate analysis, while the Student T test was used to compare the means between genders.

## Results

A total number of 1200 participants were included in our survey. Among the selected individuals, 4.75% refused to participate and were therefore replaced; age and gender characteristics between those who refused and the participants did not differ significantly (data not shown).

### Socio-demographic characteristics

Table 1 presents the socio-demographic characteristics of the study participants. The sample included 591 men (49.2%) and 609 women (50.8%). The mean age was 75.3 (SD = 7.1) years and similar in both genders. Nearly 60% of the participants were Christians, 27.7% were Muslims and 13.7% Druze. About 10% of the elderly individuals lived alone; within these, elderly women were three times more likely to live alone than men. Most of the men (84.8%), but only 44.2% of women were still married. Women were two times more likely to be illiterate than men and to suffer from worse financial status. About 46.8% of the participants had a monthly income of less than 300.000 LL and two thirds were partially or totally dependent on their children. More than 40% of the study sample did not have any health insurance. Regarding the current work-status, nearly 30% of men were still working.

**Table 1:** Socio-demographic characteristics of the study sample, distributed by gender

**Variables**	**N**^**1**^**/%**	**Total (N = 1200)**	**Males (N = 591)**	**Females (N = 609)**	**p**
**Age mean (SD)**		75.3 (7.1)	75.7 (7.2)	75.0 (6.9)	0.084
**Age Class**	**N**	**1193**	**586**	**607**	
65-75 y	%	55.0	52.9	57.0	0.353
76-85 y	%	35.0	36.9	33.3	
>85 y	%	10.0	10.2	9.7	
**Religion**	**N**	**1195**	**590**	**605**	
Christian	%	58.6	58.1	59.0	0.876
Muslims	%	27.7	27.6	27.8	
Druze	%	13.7	14.3	13.2	
**Living condition**	**N**	**1200**	**591**	**609**	
Living alone	%	9.9	4.9	14.8	<0.001
Living with others	%	90.1	95.1	85.2	
**Marital status**	**N**	**1200**	**591**	**609**	
Married	%	64.2	84.8	44.2	<0.001
Divorced/single	%	6.5	3.7	9.2	
Widowed	%	29.3	11.5	46.6	
**Education**	**N**	**1199**	**590**	**609**	
Illiterate	%	44.8	29.7	59.4	<0.001
Primary school	%	34.5	43.2	26.1	
Middle school	%	13.0	15.4	10.7	
High school/university	%	7.7	11.7	3.8	
**Monthly income**	**N**	**1143**	**571**	**572**	
< 300.000 LL	%	46.8	35.2	58.3	< 0.001
300.000 – 600.000 LL	%	26.6	29.9	23.3	
>600.000 LL	%	26.6	34.9	18.4	
**Financially dependent from children**	**N**	**1192**	**588**	**604**	
No	%	33.9	42.3	25.6	< 0.001
Partially	%	20.7	26.2	15.6	
Totally	%	45.4	31.5	58.8	
**Health Insurance**	**N**	**1194**	**588**	**606**	
No	%	41.1	38.3	43.9	0.069
Private insurance	%	5.1	6.1	4.1	
NSSF and others^2^	%	53.8	56.6	52.0	
**Currently working**	**N**	**1199**	**591**	**608**	
Yes	%	17.0	29.1	5.3	<0.001
No	**%**	83.0	70.9	94.7	
**Main occupation**	**N**	**1197**	**590**	**607**	
Farmer	%	18.5	28.0	9.4	<0.001
Employee/manager	%	25.5	41.5	9.9	
Self-employed	%	16.4	25.9	7.1	
Without work (including household)	%	39.6	4.6	73.6	

^1^Number of respondents to this question; ^2^ NSSF (national social security fund) and others.

### Nutritional status and health related characteristics

**Figure 1:**
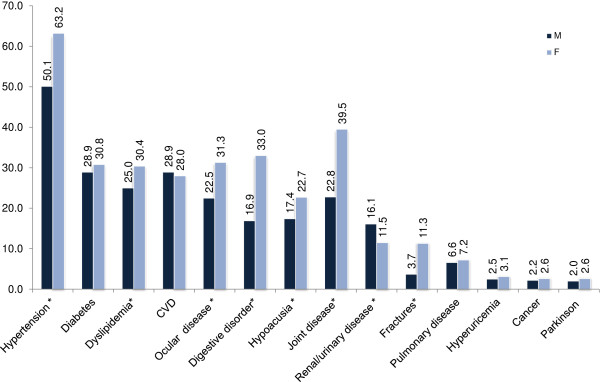
Prevalence (%) of comorbidities distributed by gender (AMEL study, N=1200).

Table 2 shows the results of the nutritional assessment and the health status of the participants. The mean BMI of our study sample was 27.5 (SD = 5.4) and was significantly higher in women than in men. Obesity was more prevalent in the female population compared to men. According to the MNA cut off points, 8.0% (95%CI 4.9% - 11.1%) of the individuals were considered as malnourished whereas 29.1% (95%CI 24.0% - 34.2%) were at risk for malnutrition. Poor nutritional status, defined as malnutrition (MN) and risk of malnutrition was significantly more frequent in females (MN: 9.1% [95%CI 4.5% - 13.7%], risk of MN: 35.3% [95%CI 28.2% - 42.6%]) than in males (MN: 6.9% [95%CI 2.8% - 11.0%], risk of MN: 22.9% [95%CI 16.1% - 29.7%]), (p < 0.001). Regarding the SRH scale, the original 5 item responses were categorized into good (good or very good), average and poor (bad or very bad). About 20% of the individuals reported poor health, whereas nearly 40% described their health as good. Women were two times more likely to consider their health as poor compared to men (p < 0.001). Comorbidity was particularly high in this population with 54.7% reporting more than three chronic diseases. The most prevalent disease was hypertension, followed by diabetes, dyslipidemia and cardiovascular disease (Figure 1). Women showed higher prevalence of the most chronic, age-related diseases (p < 0.001). Women also reported a particularly high drug intake (70.7% took more than 3 drugs daily), high frequency of chronic pain and insomnia. Considering frailty, 44.6% of elderly females, but only 28.1% of males, were considered as frail. Regarding oral health status, about two thirds of the participants, especially females, were partially or totally edentulous.

**Table 2:** Health and life-style characteristics of the study sample, distributed by gender

**Variable**	**N**^**1**^**/%**	**Total (N = 1200)**	**Males (N = 591)**	**Females (N = 609)**	**p**
**BMI** Mean (DS)		27.5 (5.4)	26.5 (4.3)	28.5 (6.1)	< 0.001
Categories	**N**	**1171**	**581**	**590**	
Underweight (<21)	%	7.6	8.4	6.8	< 0.001
Normal weight (21–24.99)	%	24.3	28.1	20.5	
Overweight (25–29.99)	%	41.1	43.2	39.0	
Obesity (≥30)	%	27.0	20.1	33.7	
**MNA** Mean (DS)		24.0 (4.3)	24.8 (4.1)	23.3 (4.4)	< 0.001
Categories	**N**	**1177**	**582**	**595**	
Malnutrition (<17)	%	8.0	6.9	9.1	< 0.001
At risk of MN (17–23.5)	%	29.1	22.9	35.3	
Normal (≥ 24)	%	62.9	70.2	55.6	
**Self related health (SRH)**	**N**	**1196**	**589**	**607**	
Good	%	38.4	45.4	31.7	< 0.001
Average	%	41.5	41.2	41.7	
Poor	%	20.1	13.4	26.6	
**Number of chronic disease**	**N**	**1199**	**591**	**608**	
>3 diseases	%	54.7	47.5	61.7	< 0.001
**Daily drug intake**	**N**	**1199**	**591**	**608**	
>3 drugs	%	62.2	53.5	70.7	< 0.001
**Chronic pain**	**N**	**1192**	**589**	**603**	
	%	45.1	35.7	54.4	< 0.001
**Insomnia**	**N**	**1198**	**590**	**608**	
No or occasionally	%	66.0	72.5	59.7	< 0.001
Often or always	%	34.0	27.5	40.3	
**SOF**	**N**	**1120**	**555**	**565**	
Robust	%	33.2	40.4	26.0	< 0.001
Prefrail	%	30.4	31.5	29.4	
Frail	%	36.4	28.1	44.6	
**Hospitalization during last year**	**N**	**1198**	**591**	**607**	
	%	45.2	41.8	48.4	0.021
**Oral health status**					
Chewing problems	**N**	**1197**	**589**	**608**	
	%	28,4	27.8	28.9	0.672
Edentulous (partially or totally)	**N**	**1095**	**545**	**550**	
	%	66.1	61.5	70.7	0.001
Dentures (partial or complete)	**N**	**1194**	**586**	**608**	
	%	47.2	41.3	52.8	< 0.001
**Physical exercise**	**N**	**1200**	**591**	**609**	
No	%	45.1	32.5	57.3	< 0.001
Occasionally	%	20.1	22.0	18.4	
Daily (≥30 min.)	%	34.8	45.5	24.3	
**Current smoker**	**N**	**1197**	**591**	**606**	
	%	25.6	32.0	19.3	< 0.001

^1^Number of respondents to this question.

### Functional ability, psycho-cognitive status and social network

Functional status of the participants is shown in Table 3. Almost 76% of the participants declared being able to perform the basic ADL. Males showed a higher level of functional ability (ADL and IADL items) compared to women. For example, regarding the IADL, males reported a 3 times higher level of independency, whereas women were two times more likely to be dependent for at least 3 IADL compared to men. Furthermore, female participants reported 1.7 times more falls than their male counterparts. In return, 61.1% of males were able to perform the “one leg balance” test but only 37.3% of females (p < 0.001). Table 3 also reports the psycho-cognitive status and social network of the study participants. Based on the 5 item GDS score, it appears that women were 1.5 times more likely to express depressive symptoms compared to men (49.6% vs 31.9%). Similar results were obtained based on the WHO-5-A score. Regarding cognitive impairment, two groups have to be considered: among the literate group (MMS1), 76.8% of the participants reached a score ≥ 24 (upper quartile), whereas in the illiterate group (MMS2) the majority of the population (68.9%) met a score of less than 24. In both groups, cognitive dysfunction was highly prevalent among women; when compared to men, women of the literate group were at least 2 times more likely to suffer from cognitive impairment compared to males. Risk of social isolation was commonly observed; in fact, nearly half of the individuals had to be considered as socially isolated (Lubben score <12). Regarding subjective loneliness, the original total score (as described by Wilson) ranging from 0 to 5, was categorized into 0–1, 2–3 and 4–5; we found that women were 1.5 times more likely to experience emotional loneliness than men (p < 0.001).

**Table 3:** Functional ability, psycho-cognitive status and social network of the study sample, distributed by gender

**Variable**	**N**^**1**^**/%**	**Total (N = 1200)**	**Males (N = 591)**	**Females (N = 609)**	**p**
**ADL**	**N**	**1199**	**591**	**608**	
Independent	%	76.0	79.5	72.5	0.015
Dependent 1 ADL	%	12.6	10.3	14.8	
Dependent for > = 2 ADL	%	11.4	10.2	12.7	
**Continence**	**N**	**1199**	**591**	**608**	
Continent	%	89.0	90.2	88.0	0.45
Partially incontinent	%	8.3	7.3	9.2	
Totally incontinent	%	2.7	2.5	2.8	
**IADL**^**2**^	**N**	**797**	**396**	**401**	
Independent	%	30.9	47.7	14.2	< 0.001
Dependent for 1 IADL	%	23.7	21.8	25.7	
Dependent for 2 IADL	%	15.8	11.9	19.7	
Dependent for 3 IADL	%	17.9	9.3	26.4	
Dependent for 4 IADL	%	11.7	9.3	14.0	
**Balance and falls**	**N**	**1197**	**590**	**607**	
One leg balance	%	49.1	61.1	37.3	< 0.001
Fall during last year	%	21.9	15.9	27.7	< 0.001
**5 item GDS score**^**3**^	**N**	**1188**	**587**	**601**	
≥ 2	%	40.8	31.9	49.6	< 0.001
**WHO-5-A score**^**4**^	**N**	**1191**	**589**	**602**	
< 13	%	53.4	38.0	55.0	< 0.001
**MMS 1**^**5**^**quartiles**	**N**	**607**	**379**	**228**	
< 24	%	23.2	16.1	35.1	< 0.001
24 - 26	%	22.6	17.9	30.3	
27 - 28	%	16.8	19.3	12.7	
> = 29	%	37.4	46.7	21.9	
**MMS 2**^**6**^**quartiles**	**N**	**516**	**182**	**334**	
< 14	%	22.9	21.4	23.7	< 0.001
14 – 19	%	23.3	13.8	28.4	
20 – 23	%	22.7	17.0	25.7	
> = 24	%	31.1	47.8	22.2	
**LUBBEN score**^**7**^	**N**	**1188**	**586**	**602**	
< 12	%	47.3	44.9	49.7	0.098
**WILSON score**^**8**^					
Mean		1.5 (1,7)	1.3 (1.6)	1.7 (1.8)	< 0.001
Categories	**N**	**1183**	**584**	**599**	
0-1	%	61,5	67.0	56.1	< 0.001
2-3	%	20,0	18.3	21.7	
4-5	%	18.5	14.7	22.2	

^1^Number of respondents to this question; ^2^IADL = Instrumental activities of daily living: missing values due to participants without telephone or with no drug intake; ^3^GDS score > =2: depression; ^4^WHO-5A- score <13: depressive disorders; ^5^MMS 1: original version; ^6^MMS 2 = adapted for illiteracy; ^7^Lubbenscore < 12: risk for social isolation; ^8^Wilson score: higher score = emotional loneliness.

### Comparison of subjects with normal cognitive status and cognitive dysfunction

When comparing sample characteristics based on cognitive status, we found important differences between the two groups (cf Table 4). Mean age was significantly higher in cognitively impaired elderly individuals (m = 76.4 y, DS = 7.2 y) compared to those with normal cognitive status (m = 73.8 y, DS = 6.6 y). In addition, significantly higher frequencies of cognitive dysfunction were found in following categories: being female (74.2%), widowed (73.9%), illiterate (86.2%), having a monthly income < 300.000 LL (71.6%), being without health insurance (61.5%). Moreover, individuals with lower occupational status had significantly lower cognitive functioning than employees, managers or those who were self-employed. Regarding nutritional status, a very high proportion of malnourished subjects (93.3%) were suffering from cognitive decline, whereas among those who had normal nutritional status, only 42.7% were found to be cognitively impaired (p < 0.001). When considering the health related variables, both mean number of chronic diseases and mean drug intake was significantly higher in cognitively impaired elderly. Furthermore cognitive dysfunction was significantly more frequent in following categories: frail elderly (74.9%), fallers (72.6%), subjects reporting hospitalization during last year (65.4%), and those reporting oral health problems. Finally, in those with the highest ADL disability (dependent for ≥ 2 ADL) and IADL disability (dependent for 4 IADL), nearly 90% showed cognitive decline (p < 0.001).

**Table 4:** Main socio-demographic and health related characteristics of the study sample distributed by cognitive status; bivariate analysis

**Variables**	**N = 1189**^**1**^	**MMS**^**2**^ **≥ 24**	**MMS < 24**	**p**
	100%	N = 517(43.5%)	N = 672(56.5%)	
**Gender**				
Male	589	61.5	38.5	<0.001
Female	600	25.8	74.2	
**Age mean (DS)**	1182	73.8 (6.6)	76.4 (7.2)	<0.001
**Religion**				
Christian	698	49.1	50.9	<0.001
Muslims	322	34.8	65.2	
Druze	164	36.0	64.0	
**Marital status**				
Married	769	52.0	48.0	<0.001
Divorced/single	75	36.0	64.0	
Widowed	345	26.1	73.9	
**Education**				
Illiterate	529	13.8	86.2	<0.001
Primary school	412	56.8	43.2	
Middle school	156	78.8	21.2	
High school/university	91	94.5	5.5	
**Monthly income**				
< 300.000 LL	528	28.4	71.6	<0.001
300.000 – 600.000 LL	304	49.3	50.7	
>600.000 LL	302	66.2	33.8	
**Health Insurance**				
No	491	38.5	61.5	<0.001
Private Insurance	60	65.0	35.0	
NSSF and others^2^	634	45.6	54.4	
**Main occupation**				
Farmer	222	37.4	62.6	<0.001
Employee/manager	304	63.2	36.8	
Self-employed	195	68.7	31.3	
Without work (including household)	465	23.0	77.0	
**BMI**	1166	27.3 (4.9)	27.8 (5.7)	0.106
**MNA**				
Malnutrition (<17)	89	6.7	93.3	<0.001
At risk of MN (17–23.5)	342	25.7	74.3	
Normal (≥ 24)	738	57.3	42.7	
**Number of chronic disease/ mean (DS)**	1188	2.5 (1.9)	3.4 (2.2)	<0.001
**Daily drug intake / mean (DS)**	1188	3.3 (2.7)	4.2(3.2)	<0.001
**Chronic pain**				
Yes	532	33.1	66.9	<0.001
No	650	52.2	47.8	
**SOF**				
Robust	370	65.1	34.9	<0.001
Prefrail	338	45.9	54.1	
Frail	406	25.1	74.9	
**Hospitalization during last year**				
Yes	535	34.6	65.4	<0.001
No	653	50.8	49.2	
**Oral health status**				
Chewing problems				
Yes	334	34.1	65.9	<0.001
No	853	47.1	52.9	
Edentulous (partially or totally)				
Yes	720	35.3	64.7	<0.001
No	365	54.0	46.0	
Dentures (partial or complete)				
Yes	562	35.9	64.1	<0.001
No	622	50.0	50.0	
**ADL**				
Independent	908	50.2	49.8	<0.001
Dependent 1 ADL	151	31.1	68.9	
Dependent for > = 2 ADL	130	10.8	89.2	
**IADL**				
Independent	246	82.5	17.5	<0.001
Dependent for 1 IADL	189	54.0	46.0	
Dependent for 2 IADL	126	39.7	60.3	
Dependent for 3 IADL	143	20.3	79.7	
Dependent for 4 IADL	89	10.1	89.9	
**Fall during last year**				
Yes	259	27.4	72.6	<0.001
No	928	48.1	51.9	
**5 item GDS**^**3**^**score mean (DS)**	1185	0.9 (1.2)	1.70 (1.5)	<0.001

^1^The total number of respondents may not add up to 1189 due to missing values; ^2^ MMS 1 (original version) and MMS 2 (adapted for illiteracy) were computed; ^3^ GDS score > =2: depressive symptoms.

## Discussion

To our knowledge this is the first study describing the living conditions, the health and nutritional status of a large representative sample of elderly Lebanese living in rural areas. This study evidenced the low socioeconomic status, the high frequency of poor nutritional and health status, and large gender disparities.

Regarding nutritional status, we found a high prevalence of malnutrition (8.0%) and risk of malnutrition (29.1%), especially in women. Our results are close to those of Kaiser et al. [20], who published pooled results from studies in five countries (Japan, South Africa, Suede, France, Switzerland) including 964 either healthy or frail home living elderly subjects (malnutrition: 5.8%, risk of malnutrition: 31.9%). Yet, the proportion of malnourished Lebanese elderly matched those reported by Guigoz among 3119 frail elderly individuals from several, mostly developed countries [19]. In a cross-sectional study conducted in Iran among community dwelling elderly people, the authors found a prevalence of 12% of malnutrition and 45.3% of risk of malnutrition [44]. In this sample, females were more often malnourished than men. In Turkey, a neighbor country of Lebanon, the risk of malnutrition, based on the MNA short from, was 28% among subjects admitted to an outpatient clinic [45]. Beside problems of malnutrition, we observed a high level of obesity and overweight, which was present in more than half of the study sample. This is typically observed in low and middle income countries, undergoing nutrition transition, which is characterized by cultural and lifestyle changes, such as decreased physical activity, shift toward more unhealthy diet patterns, modern food processing and rapid growing urbanization [46]. The coexistence of both, under – and over nutrition with a tendency toward non communicable diseases may be due to a high degree of socioeconomic inequities in these transitional countries [47]: as described by Mendez et al. [48] in a study reporting pooled data from 36 developing countries on the prevalence of over- and underweight among women, two indicators of socioeconomic development, GNI (gross national income) and urbanization, were associated with the prevalence of overweight and inversely associated with the prevalence of underweight. This double burden may result in higher prevalence of disabilities among the elderly population [2]. In our study, obesity was more prevalent among women compared to men and mean BMI was significantly higher in women despite the higher level of malnutrition. According to other authors, this may emphasize that BMI alone is not a reliable tool to assess nutritional status [49]. Our results are close to the findings from a national population-based study published by Sibai et al. [50], who reported that 27.9% of the Lebanese elderly were obese. However, this study did not provide estimates for malnutrition. Another important finding of our study is the fact that women were highly disadvantaged regarding their socioeconomic status and health. Indeed, women were significantly more often illiterate and had a lower income than men. Data from the Lebanese household survey 2007 [51] showed that among rural and urban elderly individuals, 56.0% of females versus 27.0% of males were illiterate, proportions that are very similar to those observed in our sample. Illiteracy is very common among Arabic countries [52], especially in rural areas [13]. Moreover, among females, poor nutritional status, chronic disease, frailty, functional disability and cognitive impairment were common and significantly more frequent than in men. Similar findings were reported by other authors in international and regional settings. A study conducted by Chemaitelly et al. [52] in underprivileged communities of Beirut revealed that women were less educated, reported less subjective health, more chronic diseases and functional disability. These gender differences were also described by Kabir et al. [53] in a study conducted in Bangladesh, where 80% of women reported having four or more health problems compared to only 42% of their male counterparts. These findings may be partially due to educational and cultural influences; in fact, it seems that women report more symptoms of psychological distress, anxiety, and depression than men [54]. However, women were also more disadvantaged on objective indicators such as number of drugs taken and frequency of falls. Regarding functional ability, most participants were living independently. Yet, we found that females were significantly more dependent in both, basic ADL and IADL activities. Our findings are consistent with results of previous studies conducted among older adults in Beirut [52]. These facts may be explained by the higher level of comorbidity and frailty in females. Our study also revealed that women had more balance disorders and reported significantly more falls during last year than men. Similar gender differences were present in cognitive assessment. In both versions of the MMS, women showed a significantly higher degree of cognitive impairment than men. This means that independently of literacy, women had a worse cognitive function compared to males. Higher comorbidity, depressive disorders and a higher level of loneliness [42] may explain these observations. On the other hand, it may also reflect the lack of cognitive stimulation and exercise.

Important differences were found when comparing participants according to their cognitive health (MMS < 24 and MMS ≥ 24). Thus cognitive decline was more frequent among both socially disadvantaged groups (females, illiterate and those reporting the lowest income) and subjects with lower health and functional status. These findings confirm results from previous studies reporting an association between cognitive status and several socio-demographic factors such as age [55], female gender [55,56] educational attainments [55,57,58] and having never been married [59]. Moreover, poor nutritional status [60] and functional decline [61] are both common findings in patients suffering from reduced cognitive status.

Major strengths of our study are its sample size and the rural setting. In fact, few studies focused on the specific characteristics of this population and literature shows conflicting results [62-65]. Several studies mentioned health disparities between rural and urban residents :in a study conducted in rural areas of the USA, the authors observed a higher prevalence of cardiovascular disease compared to urban areas, after controlling for possible confounders [6]. Other authors believe that these disparities are not primarily due to influence of residence but mostly to age, gender and socioeconomic status [63]. In fact, the results of two longitudinal surveys in Canada showed a strong positive relationship between socioeconomic status and health [66]. However, not only socioeconomic conditions but also a lower level of awareness may explain the rural–urban disparities. Unfortunately, in Lebanon, no comparison is possible as similar data from urban settings are not available.

Several limitations have to be considered in this survey. First, the cross-sectional design, which does not allow drawing causal relationship. Second, although our random sample can be considered as representative of the rural elderly, we could not do weighting to provide estimates of the prevalence of malnutrition for the whole Lebanese population due to missing population data in rural areas. Beside this, our sample size calculation was based on data which should not be representative for malnutrition among elderly people in developing countries. As mentioned above, this was due to lack of representative data. Furthermore, due to cognitive disorders, lack of memory and educational disparities, some information may be less accurate. In addition, some issues may affect private sphere and responses to these questions may suffer from less reliability. Moreover, most of the health related information was self-reported. Finally, several instruments were initially developed in western culture and may therefore not be culturally sensitive to Lebanon. For example, the MNA has not been validated previously in our population. Thus, it may be of great importance to undergo validation studies of this important screening tool as well as several other specific geriatric assessment tools which are not yet validated among the Lebanese population. The difficulties related to cross cultural adaptation are highlighted by el Rhazi K et al. [67] in an example of a quality of life questionnaire translated from English to Moroccan Arabic language. In the same way, several attempts were done to adapt the MMS to Arabic speaking cultures [16,68], but until now validation studies are lacking.

In terms of health and social policy, this study has several implications. First of all, rural public health programs should be implemented stressing on the importance of wellbeing of elderly people with a special focus on women’s health. Furthermore, more general practitioners, nurses and social workers have to be educated regarding health and the specific needs of the elderly population. Health care centers and home care services should be implemented and screening should focus on frail elderly and those at risk of malnutrition who may benefit from early interventions. Moreover, as socio-economic status is associated with poorer health, it is urgent to ensure pensions for elderly and to guarantee overall health insurance. Finally, a special effort should be done to equalize men and women in terms of salary and educational achievement; the latter is of great importance especially in underprivileged rural areas where illiteracy remains high and where a special effort has to be done to improve education of future generations.

## Conclusion

The present study provides unique information about the nutritional, functional and health status of community elderly people in rural areas of Lebanon. Since older adults will be an increasing part of the population, their health and well-being will also be of growing importance. It appeared that poor nutritional status (defined as malnutrition and risk of malnutrition) is frequent and concerned nearly 40% of the sample, especially women. Beside the socioeconomic disadvantage, females reported also lower self reported health and a higher level of comorbidity, drug intake, frailty, disability, depressive symptoms, loneliness and cognitive impairment. These findings may alert policy makers to plan appropriate intervention in order to improve the quality of life and increase successful aging.

## Competing interest

The authors declare that they have no competing interests.

## Authors’ contribution

CB initiated, organized and supervised the surveys, contributed to data interpretation and writing the manuscript. PS provided expertise in sampling methods, planning and organizing the survey and data analysis. PBG supervised the conception and design of the survey and the interpretation of the data. PS and PBG revised the manuscript critically. All authors read and approved the final manuscript.

## Pre-publication history

The pre-publication history for this paper can be accessed here:

http://www.biomedcentral.com/1471-2458/13/573/prepub
